# Structural features of the oxidonitridophosphates K_3_ *M* ^III^(PO_3_)_3_N (*M* ^III^ = Al, Ga)

**DOI:** 10.1107/S2056989021011336

**Published:** 2021-11-02

**Authors:** Igor V. Zatovsky, Ivan V. Ogorodnyk, Vyacheslav N. Baumer, Ivan D. Zhilyak, Ruslana V. Horda, Nataliya Yu. Strutynska

**Affiliations:** a F.D. Ovcharenko Institute of Biocolloidal Chemistry, NAS Ukraine, 42 Acad. Vernadskoho blv., 03142 Kyiv, Ukraine; b ShimUkraine LLC, 18 Chigorina Str., office 429, 01042 Kyiv, Ukraine; c STC "Institute for Single Crystals", NAS of Ukraine, 60 Lenin ave., 61001 Kharkiv, Ukraine; d Faculty of Horticulture, Ecology and Plants Protection, Uman National University of Horticulture, 1 Instytutska str., 20305 Uman, Cherkassy region, Ukraine; e Department of Inorganic Chemistry, Taras Shevchenko National University of Kyiv, 64/13, Volodymyrska str., 01601, Kyiv, Ukraine

**Keywords:** crystal structure, isotypism, (PO_3_)_3_N anion, cubic symmetry, self-flux crystal growth

## Abstract

Cubic crystals of tripotassium aluminium (or gallium) nitridotriphosphate, K_3_ *M* ^III^(PO_3_)_3_N (*M* ^III^ = Al, Ga), were grown by application of the self-flux method. In their isostructural crystal structures, all metal cations and the N atom occupy special positions with site symmetry 3, while the P and O atoms are situated in general positions. The three-dimensional framework of these oxidonitridophosphates is built up from [*M* ^III^O_6_] octa­hedra linked together *via* (PO_3_)_3_N groups. The latter are formed from three PO_3_N tetra­hedra sharing a common N atom. The coordination environments of the three potassium cations are represented by two types of polyhedra, *viz*. KO_9_ for one and KO_9_N for the other two cations. An unusual tetra­dentate type of coordination for the latter potassium cations by the (PO_3_)_3_N^6–^ anion is observed. These K_3_ *M* ^III^(PO_3_)_3_N (*M* ^III^ = Al, Ga) compounds are isostructural with the Na_3_ *M* ^III^(PO_3_)_3_N (*M* ^III^ = Al, V, Ti) compounds.

## Abstract 2

Cubic K_3_ *M* ^III^(PO_3_)_3_N (*M* ^III^ = Al, Ga) is isostructural with (*M* ^III^ = Al, V, Ti). In the potassium compounds, the (PO_3_)_3_N^6–^ anion coordinates in a tetra­dentate manner to two potassium cations.

## Chemical context

Oxidonitridophosphates with general compositions *M* ^I^_3_ *M* ^III^(PO_3_)_3_N and *M* ^I^_2_ *M* ^II^_2_(PO_3_)_3_N (*M* ^I^ = Li, Na, K; *M* ^III^ = Al, Cr, Ga, V and Ti; *M* ^II^ = Mg, Fe) have been prepared by solid-state synthesis (Feldmann, 1987*a* ▸,*b* ▸; Massiot *et al.*, 1996 ▸; Conanec *et al.*, 1996 ▸), high-temperature thermal ammonolysis (Kim & Kim, 2013 ▸; Kim *et al.*, 2017 ▸; Zhang *et al.*, 2017 ▸), flux-growth (Zatovsky *et al.*, 2006 ▸) or solid–solid ion-exchange (Liu *et al.*, 2018 ▸). In recent years, oxidonitridophosphates containing the (PO_3_)_3_N^6–^ anion have been intensively studied as promising cathode materials for Na-ion and Li-ion batteries. In particular, ionic conductivities and redox properties were investigated in detail for Na_3_Ti(PO_3_)_3_N (Liu *et al.*, 2014 ▸), Na_3_V(PO_3_)_3_N (Reynaud *et al.*, 2017 ▸; Kim *et al.*, 2017 ▸; Zhang *et al.*, 2017 ▸; Xiao *et al.*, 2021 ▸; Wang *et al.*, 2021 ▸), Li_3_V(PO_3_)_3_N (Liu *et al.*, 2018 ▸), Na_2_Fe_2_(PO_3_)_3_N and Li_2_Fe_2_(PO_3_)_3_N (Liu *et al.*, 2013 ▸), Na_2_Mg_2_(PO_3_)_3_N (Cosby *et al.*, 2020 ▸), and Li_2_Mg_2_(PO_3_)_3_N (Liu *et al.*, 2017 ▸). In addition, rare-earth-doped Na_3_Al(PO_3_)_3_N has been studied as a promising phosphor (Bang *et al.*, 2013 ▸). However, the structural data for oxidonitridophosphates include only Na- or Li-containing compounds (Massiot *et al.*, 1996 ▸; Zatovsky *et al.*, 2006 ▸; Kim & Kim, 2013 ▸; Liu *et al.*, 2013 ▸, 2017 ▸, 2018 ▸; Zhang *et al.*, 2017 ▸).

In this communication, we report the flux-growth synthesis, structural characterization and FTIR spectra for the two K-containing oxidonitridophosphates K_3_Al(PO_3_)_3_N (I) and K_3_Ga(PO_3_)_3_N (II).

## Structural commentary

**Figure 1:**
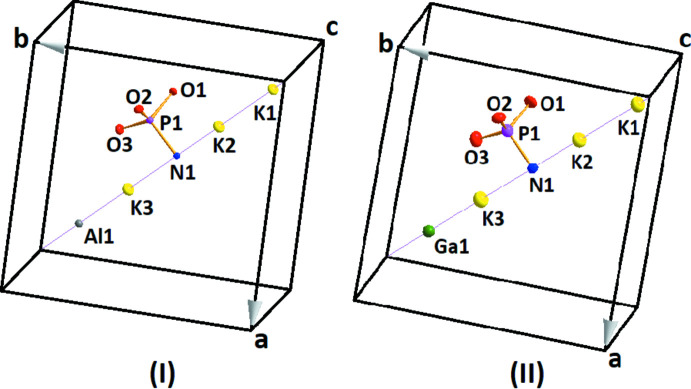
A view of the asymmetric units of K_3_Al(PO_3_)_3_N (I) and K_3_Ga(PO_3_)_3_N (II), with displacement ellipsoids drawn at the 50% probability level.

**Figure 2:**
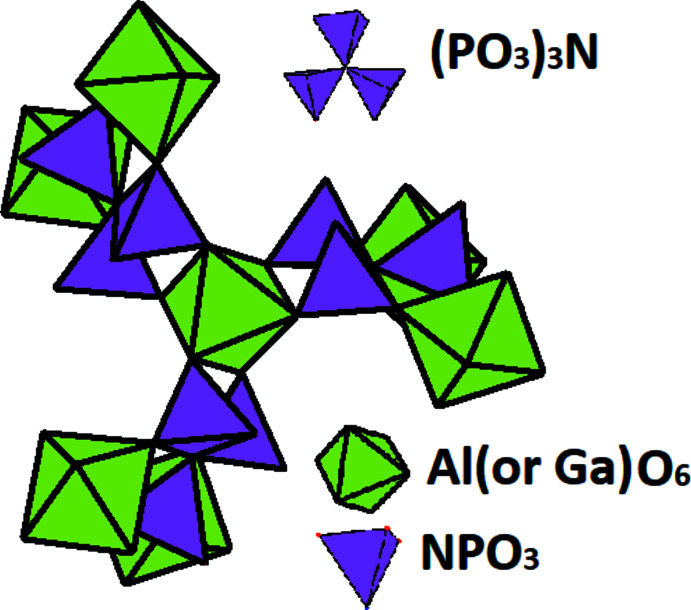
[*M* ^III^O_6_] octa­hedra and ‘three-blade propeller’-type anions (PO_3_)_3_N^6–^ as principle building units for formation of the three-dimensional framework of (I) and (II).

Compounds (I) and (II) (Fig. 1 ▸) are isotypic and crystallize in the cubic Na_3_Al(PO_3_)_3_N structure in space-group type *P*2_1_3. The K, Al (or Ga) and N atoms are localized on threefold rotation axes (Wyckoff position 4 *a*), and the P and all O atoms occupy general 12 *b* sites (Fig. 1 ▸). As shown in Fig. 2 ▸, the crystal structures of the K_3_ *M* ^III^(PO_3_)_3_N (*M* ^III^ = Al, Ga) title compounds consist of [*M* ^III^O_6_] octa­hedra and (PO_3_)_3_N^6–^ anions, which are linked *via* vertices, forming a three-dimensional framework. The (PO_3_)_3_N^6–^ anion is built up from three PO_3_N tetra­hedra sharing a common N vertex atom. The P—O bond lengths for both structures range between 1.473 (9) and 1.534 (3) Å (Tables 1 ▸ and 2 ▸), while the P—N bond lengths are 1.7084 (12) and 1.701 (4) Å for (I) and (II), respectively. The lengths of the P—O and P—N bonds are similar to those found in the isotypic oxidonitridophosphates such as Na_3_Al(PO_3_)_3_N (Conanec *et al.*, 1994 ▸, 1996 ▸), Na_3_Ti(PO_3_)_3_N (Zatovsky *et al.*, 2006 ▸), or Na_3_V(PO_3_)_3_N (Kim & Kim, 2013 ▸). The octa­hedral coordination environments around *M* ^III^ are slightly distorted, as indicated by the different *M* ^III^—O bond lengths (Tables 1 ▸ and 2 ▸). The [*M* ^III^O_6_] octa­hedra are slightly squeezed along the cubic cell diagonal. The average Al—O and Ga—O distances are 1.907 (3) and 1.963 (9) Å, respectively. These values are close to the sums of the ionic radii (Shannon, 1976 ▸) of Al^3+^ and O^2–^ (1.92 Å) and Ga^3+^ and O^2−^ (2.00 Å), respectively.

**Figure 3:**
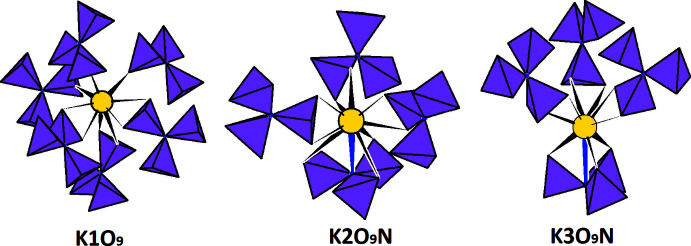
The coordination environment of potassium cations in (I) and (II).

**Figure 4:**
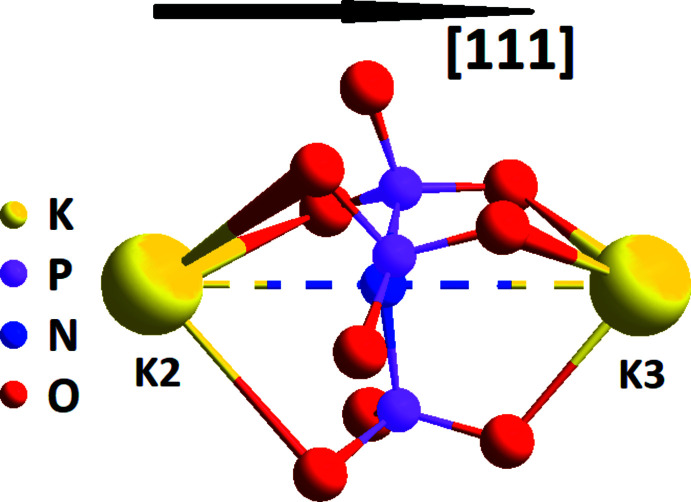
Coordination of K2 and K3 cations by the (PO_3_)_3_N^6–^ anion for (I) and (II).

Fig. 3 ▸ shows the coordination environments of potassium cations for (I) and (II). K1 has nine O-atom neighbors with K—O distances ranging from 2.623 (3) to 3.261 (3) Å, which includes three mono- and three bidentately coordinating (PO_3_)_3_N^6–^ anions. K2O_9_N and K3O_9_N polyhedra are formed as a result of one tetra- and three bidentately coordinating oxidonitridophosphate anions. In the latter case, the upper boundary for K—O distances is 3.412 (8) Å; K2—N distances are 2.904 (6) and 2.977 (18) Å and K3—N contacts are 3.340 (6) and 3.310 (18) Å for (I) and (II), respectively. The coordination environments around the alkali metal for K-containing oxidonitridophosphates (K2O_9_N and K3O_9_N polyhedra) differ from those of the Na-containing compounds (Na2O_6_N and Na3O_6_N polyhedra). In addition, the (PO_3_)_3_N^6–^ anions coordinate the two potassium cations in a tetra­dentate manner (Fig. 4 ▸). As is shown schematically in Fig. 4 ▸ and in Table 3 ▸ for the isotypic *M* ^I^_3_ *M* ^III^(PO_3_)_3_N compounds, the *M* ^I^, *M* ^III^ and N atoms are arranged along the [111] direction in the sequence –*M*1^I^–*M*2^I^–N–*M*3^I^–*M* ^III^–*M*1^I^– whereby the *M*2^I^—N—*M*3^I^ distances change in a different manner. In case of (I) and (II), the shape of the (PO_3_)_3_N^6–^ anion is similar (the P—N distances are about 1.70 Å and the P—N—K3 angles are within 83-84°; Tables 1 ▸ and 2 ▸). For the Na-containing analogues, the P—N bond is slightly larger (1.71–1.74 Å) and the P—N—Na3 angles are smaller within a wider range from 75 to 78° (Conanec *et al.*, 1994 ▸, 1996 ▸; Zatovsky *et al.*, 2006 ▸; Kim & Kim, 2013 ▸).

To clarify some points regarding the structural changes we have calculated the bond-valence sums (BVS) for the K, P and Al atoms (I) and Ga atoms (II), respectively. Parameters were taken from Brown & Altermatt (1985 ▸) and for the K—N bond from Brese & O’Keeffe (1991 ▸). The BVS for positively charged atoms in (I) is 21.64 v.u. and 22.28 v.u. for (II) while the sum of the charges of nine O and one N atom is equal to 21 v.u. (Table 4 ▸). The higher values for the Ga-containing compound might be explained as follows. The BVS for Al in [AlO_6_] was found to be 3.00 v.u. while for Ga in [GaO_6_] it is 3.20 v.u. The remaining atoms also show a slight overbonding (Table 4 ▸). We suppose that the anionic part (PO_3_)_3_N^6–^ is rigid enough and cannot be stretched to larger sizes relative to the larger [GaO_6_] octa­hedron into a more expanded framework. This is the reason why shorter inter­atomic K—O and P—O distances are observed in the structure of (II) compared to that of (I) (Tables 1 ▸ and 2 ▸). As expected, the unit-cell parameters of (I) are smaller than for (II), in good agreement with the ionic radii of Al^3+^ and Ga^3+^ (Shannon, 1976 ▸). In other words, the rigid and almost flat ‘three-blade propeller’ anions combine with [*M* ^III^O_6_] octa­hedra to form the framework in which the cavities for the alkali cations become smaller as greater octa­hedra are involved. Moreover, the greater [*M* ^III^O_6_] octa­hedra strongly influence the ‘three-blade propeller’ anion, resulting in slightly shorter P—O and P—N bonds. On the other hand, the local environments of the K cations should also be mentioned. In the K2O_9_N polyhedron, the BVS for K2 is 1.12 v.u. in (I) and 1.07 in (II). The contribution to the K2—N1 bond to the valence sum is 0.12 v.u. for (I) and 0.10 for (II). These high values indicate a strong inter­action between the two atoms. The cavities in which K1 and K2 are located become larger with longer or almost the same K⋯O and K⋯N contacts, while the cavities in which K3 is situated become smaller with shortened K⋯O contacts in (II) in comparison with (I). BVS calculations for the phosphate tetra­hedra show similar results (Table 4 ▸).

It should also be noted that further theoretical calculations of the electronic structure can bring final clarity to the principles of bonding of alkali cations with (PO_3_)_3_N^6–^ anions in K_3_ *M* ^III^(PO_3_)_3_N compounds. This could be a way for the creation of new materials with desired properties based on K-containing oxidonitridophosphates.

## Synthesis and crystallization

For the synthesis of (I) and (II), KH_2_PO_4_, K_4_P_2_O_7_·10H_2_O, urea, Al_2_O_3_ or Ga_2_O_3_ (all analytically or extra pure grade) were used as initial reagents. The sequence of preparation procedure was as follows: (1) phosphates KPO_3_ and K_4_P_2_O_7_ were each prepared by calcining KH_2_PO_4_ and K_4_P_2_O_7_·10H_2_O at 873 K; (2) a mixture of 20.07 g of KPO_3_, 13.21 g of K_4_P_2_O_7_ and 30.03 g of urea (molar ratio K:P = 1:1.32, urea:P = 2:1) ground in an agate mortar, was heated to complete degassing at 623 K in a porcelain dish. The resulting solid was reground and heated to become a homogeneous liquid at 1023 K and then quenched by pouring the melt onto a copper sheet to form a glass. The glass was crushed using a mill, and a powder with a particle size of less than 125 µm was separated. According to chemical analysis, the prepared glass had the composition K_1.32_PO_2.43_N_0.50_; (3) a mixture of 10 g of glass (K_1.32_PO_2.43_N_0.50_) and 0.3 g of Al_2_O_3_ or 0.7 g of Ga_2_O_3_ powders were placed into porcelain crucibles and heated up to 1043 K and then cooled to 923 K at a rate of 25 K h^−1^. After cooling to room temperature, colorless tetra­hedral crystals of (I) or (II) were washed with deionized water. Elemental analysis indicated the presence of K, Al (or Ga), P and N in the atomic ratio 3:1:3:1.

The growing of well-shaped crystals of (I) and (II) suitable for single crystal X-ray diffraction analysis was one of main tasks during the present study. Hence, a similar way for the preparation of the potassium-containing phosphates to that for the previously reported sodium-containing compounds was applied, following the self-flux method for the preparation of crystalline nitro­gen-containing phosphates (Zatovsky *et al.*, 2006 ▸). Thermal decomposition of urea is a multistage process and leads to the formation of C_3_N_4_ (Wang *et al.*, 2017 ▸). The initial *M* ^I^–P–O–N (*M* ^I^ = alkali metal) melt can be obtained by the reaction of urea with alkali metal phosphates, when a mixture of phosphates and C_3_N_4_ inter­act. The change of the phosphate:C_3_N_4_ ratio (or phosphate:urea) and the nature of the alkali metal affects the composition of the resulting melt. In our case, the molar ratio of K:P was chosen to be 1:1.32 because a mixture of KPO_3_ and K_4_P_2_O_7_ in this ratio has the lowest melting point close to 886 K, and the urea:P ratio was set to 2:1. As a result, a glass of composition K_1.32_PO_2.43_N_0.50_ was obtained.

The solubilities of Al_2_O_3_ and Ga_2_O_3_ in the K_1.32_PO_2.43_N_0.50_ self-fluxes differ significantly. Crystallization of compound (I) occurs as a result of the inter­action of self-fluxes and 2–4%wt. Al_2_O_3_. The formation of a mixture of (I) and Al_2_O_3_ was observed when the initial amount of aluminum oxide was higher than 5%wt. The solubility of Ga_2_O_3_ in the self-flux is about 7%wt. at 1043 K, and subsequent cooling of the homogeneous melt led to the crystallization of compound (II). In all cases, the amount of nitro­gen in the self-fluxes rapidly decreases above 1063 K. This process occurs due to the thermal instability of P—N bonds at high temperatures, and leads to a redox reaction with the release of nitro­gen and phospho­rus. The latter vaporizes from the phosphate melts and starts to burn, which can be observed by periodical sparks on the melt surface (Zatovsky *et al.*, 2000 ▸). As a result, K–*M* ^III^–P–O (*M* ^III^ = Al, Ga) melts prone to vitrifying are formed.

**Figure 5:**
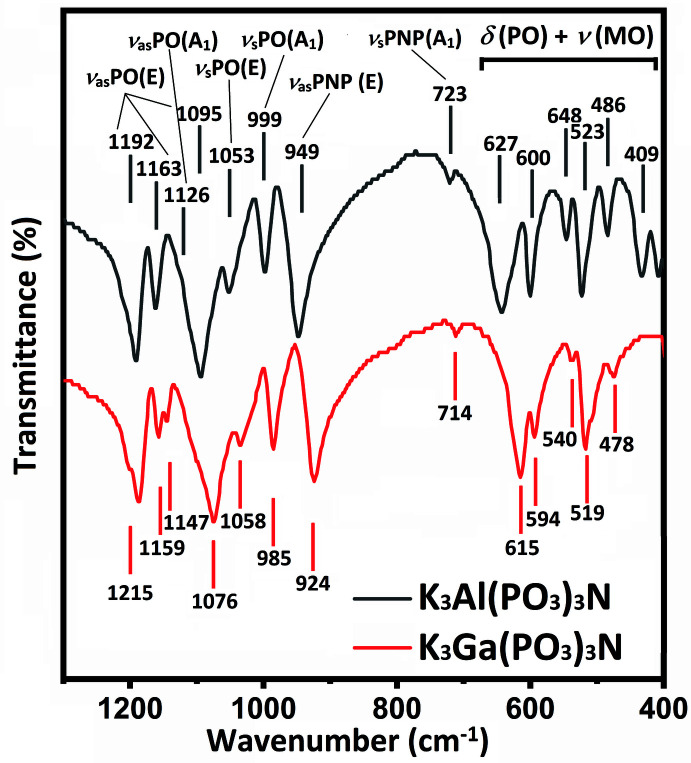
FTIR spectra of (I) and (II).

The obtained compounds (I) and (II) were further characterized using FTIR spectroscopy; FTIR spectra were collected at room temperature on KBr discs using a Thermo NICOLET Nexus 470 spectrophotometer. As can be seen in Fig. 5 ▸, the spectra of (I) and (II) are similar with respect to intensities and band positions (the difference in the band positions does not exceed 27 cm^−1^). The characteristic bands are in good agreement with the presence of the N(PO_3_)_3_^6–^ anion with *C*_3*v*_ symmetry, which provides for a set of vibration modes: 6A1 + 5*A*2 + 11*E* (3*A*1 + *A*2 + 4*E* belong to stretching vibrations, and 3*A*1 + 4*A*2 + 7*E* are due to deformation vibrations). As shown in Fig. 5 ▸, the following regions can be distinguished in the FTIR spectra: (1) the bands in the region between 980 and 1220 cm^−1^ are assigned to ν_as_ and ν_s_(PO_3_) stretching vibrations [four absorption bands in the frequency range between 1070 and 1220 cm^−1^ belong to *ν*_as_(P—O), and two bands of between 980 and 1060 cm^−1^ correspond to ν_s_(P—O)]; (2) ν_as_(P—N—P) and ν_s_(P—N—P) bands can be observed in the regions between 920 and 950 cm^−1^ and around 920 cm^−1^, respectively; (3) the range between 400 and 680 cm^−1^ includes absorption bands due to δ (P—O) and ν (Al—O) or ν (Ga—O) vibrations. In summary, in terms of the set of absorption bands, the FTIR spectra of the N(PO_3_)_3_^6–^ anion resemble those of the P_2_O_7_^4–^ anion.

## Refinement

Crystal data, data collection and structure refinement details are summarized in Table 5 ▸. As a result of the shapes of the obtained crystals, their cell parameters and chemical compositions, we had expected that both structures should be isostructural with the previously reported Na_3_Al(PO_3_)_3_N and Na_3_Ti(PO_3_)_3_N structures. In fact, analysis of the single-crystal data showed that both compounds crystallize in the same space group type (*P*2_1_3) as the Na-containing oxidonitridophosphates. Originally, the crystal structures were solved by direct methods but we also performed refinements using the atomic coordinates of Na_3_Ti(PO_3_)_3_N as a starting model. The results were the same, confirming that both structures are isostructural with Na_3_Ti(PO_3_)_3_N (as well as with all previously reported cubic oxionitridophosphates with the same formula type).

## Supplementary Material

Crystal structure: contains datablock(s) global, I, II. DOI: 10.1107/S2056989021011336/wm5621sup1.cif

Structure factors: contains datablock(s) I. DOI: 10.1107/S2056989021011336/wm5621Isup2.hkl

Structure factors: contains datablock(s) II. DOI: 10.1107/S2056989021011336/wm5621IIsup3.hkl

CCDC references: 2118192, 2118191

Additional supporting information:  crystallographic information; 3D view; checkCIF report

## Figures and Tables

**Table 1:** Selected geometric parameters (Å, °) for (I)

K1—O3^i^	2.623 (3)	K3—N1	3.340 (6)
K1—O1^ii^	2.761 (3)	K3—O3^iv^	3.379 (3)
K1—O2^ii^	3.261 (3)	Al1—O1^v^	1.905 (3)
K2—O3^iii^	2.765 (3)	Al1—O2^iv^	1.909 (3)
K2—N1	2.904 (6)	P1—O3	1.493 (3)
K2—O1^iii^	2.967 (3)	P1—O1	1.530 (3)
K2—O2	3.263 (3)	P1—O2	1.534 (3)
K3—O3	2.679 (3)	P1—N1	1.7084 (12)
K3—O2^iv^	2.803 (3)		
			
O3—P1—O1	111.58 (17)	O1—P1—N1	105.82 (13)
O3—P1—O2	113.45 (16)	O2—P1—N1	105.20 (17)
O1—P1—O2	109.85 (16)	P1^vi^—N1—P1	118.53 (8)
O3—P1—N1	110.5 (2)		

Symmetry codes: (i) -y+1, z-{\script{1\over 2}}, -x+{\script{1\over 2}}; (ii) -z+{\script{1\over 2}}, -x, y-{\script{1\over 2}}; (iii) z-{\script{1\over 2}}, -x+{\script{1\over 2}}, -y+1; (iv) -z+1, x+{\script{1\over 2}}, -y+{\script{3\over 2}}; (v) -z+{\script{3\over 2}}, -x+1, y+{\script{1\over 2}}; (vi) z, x, y.

**Table 2:** Selected geometric parameters (Å, °) for (II)

K1—O3^i^	2.642 (8)	K3—N1	3.310 (18)
K1—O1^ii^	2.807 (9)	K3—O3^v^	3.412 (8)
K1—O2^iii^	3.216 (8)	Ga1—O2^vi^	1.968 (9)
K2—O3^iv^	2.808 (9)	Ga1—O2^vii^	1.968 (9)
K2—O1^i^	2.925 (9)	P1—O3	1.473 (9)
K2—N1	2.977 (18)	P1—O2	1.516 (9)
K2—O2	3.287 (8)	P1—O1	1.524 (9)
K3—O3	2.665 (9)	P1—N1	1.701 (4)
K3—O2^v^	2.785 (8)		
			
O3—P1—O2	113.8 (5)	O2—P1—N1	107.0 (5)
O3—P1—O1	112.1 (5)	O1—P1—N1	106.8 (4)
O2—P1—O1	107.9 (5)	P1—N1—P1^viii^	118.8 (2)
O3—P1—N1	108.9 (7)		

Symmetry codes: (i) z-{\script{1\over 2}}, -x+{\script{1\over 2}}, -y+1; (ii) -x, y-{\script{1\over 2}}, -z+{\script{1\over 2}}; (iii) -z+{\script{1\over 2}}, -x, y-{\script{1\over 2}}; (iv) -y+1, z-{\script{1\over 2}}, -x+{\script{1\over 2}}; (v) -z+1, x+{\script{1\over 2}}, -y+{\script{3\over 2}}; (vi) -y+{\script{3\over 2}}, -z+1, x+{\script{1\over 2}}; (vii) x+{\script{1\over 2}}, -y+{\script{3\over 2}}, -z+1; (viii) z, x, y.

**Table 3:** BVS results (v.u.) for (I) and (II)

Central Atom	(I)	(II)
Al1/Ga1	3.004	3.197
K1	1.486	1.400
K2	1.122	1.069
K3	1.315	1.360
P1	3 × 4.903	3 × 5.084
Σ	21.636	22.278

**Table 4:** Distances (Å) between atoms for (I), (II) and isotypic *M* ^I^_3_ *M* ^III^(PO_3_)_3_N compounds along [111]

Compound	Atomic distance between neighboring atoms	Reference
Na_3_Al(PO_3_)_3_N	–Na1–3.438–Na2–2.875–N–3.197–Na3–3.068–Al–3.486–Na1–	Massiot *et al.* (1996 ▸)
Na_3_Ti(PO_3_)_3_N	–Na1–3.448–Na2–3.078–N–3.188–Na3–3.100–Ti–3.638–Na1–	Zatovsky *et al.* (2006 ▸)
Na_3_V(PO_3_)_3_N	–Na1–3.477–Na2–2.947–N–3.234–Na3–3.100–V–3.606–Na1–	Kim & Kim (2013 ▸)
K_3_Al(PO_3_)_3_N	–K1–3.723–K2–2.904–N–3.340–K3–3.400–Al–3.429–K1–	This work
K_3_Ga(PO_3_)_3_N	–K1–3.747–K2–2.978–N–3.310–K3–3.350–Ga–3.470–K1–	This work

**Table 5:** Experimental details

	(I)	(II)
Crystal data		
Chemical formula	K_3_Al(NP_3_O_9_)	K_3_Ga(NP_3_O_9_)
*M*_r_	395.2	437.94
Crystal system, space group	Cubic, *P*2_1_3	Cubic, *P*2_1_3
Temperature (K)	293	293
*a* (Å)	9.6970 (4)	9.7313 (9)
*V* (Å^3^)	911.83 (11)	921.5 (3)
*Z*	4	4
Radiation type	Mo *K*α	Mo *K*α
μ (mm^−1^)	2.16	4.90
Crystal size (mm)	0.15 × 0.12 × 0.1	0.12 × 0.07 × 0.04

Data collection		
Diffractometer	Oxford Diffraction Xcalibur-3	Oxford Diffraction Xcalibur-3
Absorption correction	Multi-scan (Blessing, 1995 ▸)	Multi-scan (Blessing, 1995 ▸)
*T*_min_, *T*_max_	0.844, 0.869	0.738, 0.804
No. of measured, independent and observed [*I* > 2σ(*I*)] reflections	7172, 745, 667	4964, 753, 517
*R*_int_	0.078	0.155
(sin θ/λ)_max_ (Å^−1^)	0.660	0.660

Refinement		
*R*[*F* ^2^ > 2σ(*F* ^2^)], *wR*(*F* ^2^), *S*	0.026, 0.052, 1.05	0.059, 0.102, 1
No. of reflections	745	753
No. of parameters	53	53
Δρ_max_, Δρ_min_ (e Å^−3^)	0.27, −0.27	0.67, −0.60
Absolute structure	Flack *x* determined using 269 quotients [(*I* ^+^)−(*I* ^−^)]/[(*I* ^+^)+(*I* ^−^)] (Parsons *et al.*, 2013 ▸)	Flack *x* determined using 153 quotients [(*I* ^+^)−(*I* ^−^)]/[(*I* ^+^)+(*I* ^−^)] (Parsons *et al.*, 2013 ▸)
Absolute structure parameter	−0.02 (6)	0.03 (5)

Computer programs: *CrysAlis CCD* (Oxford Diffraction, 2006 ▸), *CrysAlis RED* (Oxford Diffraction, 2006 ▸), *SHELXS* (Sheldrick, 2008 ▸), *SHELXS2013/1* (Sheldrick, 2008 ▸), *SHELXL2018/3* (Sheldrick, 2015 ▸), *DIAMOND* (Brandenburg, 2006 ▸), *WinGX* (Farrugia, 2012 ▸), *enCIFer* (Allen *et al.*, 2004 ▸) and *publCIF* (Westrip, 2010 ▸).

## supplementary crystallographic information

### Tripotassium aluminium nitridotriphosphate (I) Crystal data

**Table:**

K_3_Al(NP_3_O_9_)	*D*_x_ = 2.879 Mg m^−^^3^
*M**_r_* = 395.2	Mo *K*α radiation, λ = 0.71073 Å
Cubic, *P*2_1_3	Cell parameters from 7172 reflections
Hall symbol: P 2ac 2ab 3	θ = 3.0–28.0°
*a* = 9.6970 (4) Å	µ = 2.16 mm^−^^1^
*V* = 911.83 (11) Å^3^	*T* = 293 K
*Z* = 4	Tetrahedron, colorless
*F*(000) = 776	0.15 × 0.12 × 0.1 mm

### Tripotassium aluminium nitridotriphosphate (I) Data collection

**Table:**

Oxford Diffraction Xcalibur-3 diffractometer	667 reflections with *I* > 2σ(*I*)
Graphite monochromator	*R*_int_ = 0.078
φ and ω scans	θ_max_ = 28.0°, θ_min_ = 3.0°
Absorption correction: multi-scan (Blessing, 1995)	*h* = −12→12
*T*_min_ = 0.844, *T*_max_ = 0.869	*k* = −12→10
7172 measured reflections	*l* = −12→12
745 independent reflections	

### Tripotassium aluminium nitridotriphosphate (I) Refinement

**Table:**

Refinement on *F*^2^	'*w* = 1/[σ^2^(*F*_o_^2^) + (0.0211*P*)^2^] where *P* = (*F*_o_^2^ + 2*F*_c_^2^)/3'
Least-squares matrix: full	(Δ/σ)_max_ < 0.001
*R*[*F*^2^ > 2σ(*F*^2^)] = 0.026	Δρ_max_ = 0.27 e Å^−^^3^
*wR*(*F*^2^) = 0.052	Δρ_min_ = −0.27 e Å^−^^3^
*S* = 1.05	Extinction correction: SHELXL2018/3 (Sheldrick, 2015)
745 reflections	Extinction coefficient: 0.0037 (14)
53 parameters	Absolute structure: Flack *x* determined using 269 quotients [(*I*^+^)-(*I*^-^)]/[(*I*^+^)+(*I*^-^)] (Parsons et al., 2013)
0 restraints	Absolute structure parameter: −0.02 (6)

### Tripotassium aluminium nitridotriphosphate (I) Special details

**Table:**

Geometry. All esds (except the esd in the dihedral angle between two l.s. planes) are estimated using the full covariance matrix. The cell esds are taken into account individually in the estimation of esds in distances, angles and torsion angles; correlations between esds in cell parameters are only used when they are defined by crystal symmetry. An approximate (isotropic) treatment of cell esds is used for estimating esds involving l.s. planes.

### Tripotassium aluminium nitridotriphosphate (I) Fractional atomic coordinates and isotropic or equivalent isotropic displacement parameters (Å^2^)

**Table:**

	*x*	*y*	*z*	*U*_iso_*/*U*_eq_	
K1	0.04655 (9)	0.04655 (9)	0.04655 (9)	0.0178 (4)	
K2	0.26820 (10)	0.26820 (10)	0.26820 (10)	0.0195 (4)	
K3	0.63996 (10)	0.63996 (10)	0.63996 (10)	0.0207 (4)	
Al1	0.84238 (12)	0.84238 (12)	0.84238 (12)	0.0095 (5)	
P1	0.32280 (11)	0.56862 (11)	0.46909 (11)	0.0088 (3)	
N1	0.4411 (3)	0.4411 (3)	0.4411 (3)	0.0073 (11)	
O1	0.2129 (3)	0.5055 (3)	0.5631 (3)	0.0107 (6)	
O2	0.2606 (3)	0.5994 (3)	0.3269 (3)	0.0120 (6)	
O3	0.3898 (3)	0.6909 (3)	0.5344 (3)	0.0161 (7)	

### Tripotassium aluminium nitridotriphosphate (I) Atomic displacement parameters (Å^2^)

**Table:**

	*U*^11^	*U*^22^	*U*^33^	*U*^12^	*U*^13^	*U*^23^
K1	0.0178 (4)	0.0178 (4)	0.0178 (4)	−0.0037 (4)	−0.0037 (4)	−0.0037 (4)
K2	0.0195 (4)	0.0195 (4)	0.0195 (4)	−0.0034 (4)	−0.0034 (4)	−0.0034 (4)
K3	0.0207 (4)	0.0207 (4)	0.0207 (4)	−0.0040 (4)	−0.0040 (4)	−0.0040 (4)
Al1	0.0095 (5)	0.0095 (5)	0.0095 (5)	−0.0006 (5)	−0.0006 (5)	−0.0006 (5)
P1	0.0081 (5)	0.0087 (6)	0.0096 (5)	0.0003 (4)	0.0010 (4)	0.0003 (4)
N1	0.0073 (11)	0.0073 (11)	0.0073 (11)	0.0001 (13)	0.0001 (13)	0.0001 (13)
O1	0.0103 (15)	0.0108 (14)	0.0110 (14)	0.0004 (11)	0.0043 (12)	0.0021 (12)
O2	0.0116 (15)	0.0147 (15)	0.0099 (14)	0.0022 (12)	−0.0019 (12)	0.0007 (12)
O3	0.0163 (16)	0.0116 (16)	0.0204 (16)	−0.0023 (12)	−0.0019 (14)	−0.0037 (13)

### Tripotassium aluminium nitridotriphosphate (I) Geometric parameters (Å, º)

**Table:**

K1—O3^i^	2.623 (3)	K2—P1^ii^	3.4199 (12)
K1—O3^ii^	2.623 (3)	K3—O3^viii^	2.679 (3)
K1—O3^iii^	2.623 (3)	K3—O3^ix^	2.679 (3)
K1—O1^iv^	2.761 (3)	K3—O3	2.679 (3)
K1—O1^v^	2.761 (3)	K3—O2^x^	2.803 (3)
K1—O1^vi^	2.761 (3)	K3—O2^xi^	2.803 (3)
K1—O2^iv^	3.261 (3)	K3—O2^xii^	2.803 (3)
K1—O2^vi^	3.261 (3)	K3—N1	3.340 (6)
K1—O2^v^	3.261 (3)	K3—O3^x^	3.379 (3)
K1—Al1^vii^	3.429 (3)	K3—O3^xi^	3.379 (3)
K1—P1^iv^	3.5911 (13)	K3—O3^xii^	3.379 (3)
K1—P1^vi^	3.5911 (13)	K3—Al1	3.400 (3)
K2—O3^iii^	2.765 (3)	K3—P1^xii^	3.4997 (14)
K2—O3^i^	2.765 (3)	Al1—O1^xiii^	1.905 (3)
K2—O3^ii^	2.765 (3)	Al1—O1^xiv^	1.905 (3)
K2—N1	2.904 (6)	Al1—O1^xv^	1.905 (3)
K2—O1^iii^	2.967 (3)	Al1—O2^x^	1.909 (3)
K2—O1^i^	2.967 (3)	Al1—O2^xi^	1.909 (3)
K2—O1^ii^	2.967 (3)	Al1—O2^xii^	1.909 (3)
K2—O2	3.263 (3)	P1—O3	1.493 (3)
K2—O2^viii^	3.263 (3)	P1—O1	1.530 (3)
K2—O2^ix^	3.263 (3)	P1—O2	1.534 (3)
K2—P1^iii^	3.4199 (12)	P1—N1	1.7084 (12)
			
O3^i^—K1—O3^ii^	79.95 (10)	O2^x^—K3—O3^xi^	60.45 (8)
O3^i^—K1—O3^iii^	79.95 (10)	O2^xi^—K3—O3^xi^	47.20 (7)
O3^ii^—K1—O3^iii^	79.95 (10)	O2^xii^—K3—O3^xi^	99.58 (8)
O3^i^—K1—O1^iv^	111.53 (9)	N1—K3—O3^xi^	113.91 (5)
O3^ii^—K1—O1^iv^	165.73 (9)	O3^x^—K3—O3^xi^	104.69 (6)
O3^iii^—K1—O1^iv^	109.72 (8)	O3^viii^—K3—O3^xii^	143.68 (7)
O3^i^—K1—O1^v^	109.72 (8)	O3^ix^—K3—O3^xii^	66.44 (10)
O3^ii^—K1—O1^v^	111.53 (9)	O3—K3—O3^xii^	111.63 (2)
O3^iii^—K1—O1^v^	165.73 (9)	O2^x^—K3—O3^xii^	99.58 (8)
O1^iv^—K1—O1^v^	57.44 (9)	O2^xi^—K3—O3^xii^	60.45 (8)
O3^i^—K1—O1^vi^	165.73 (9)	O2^xii^—K3—O3^xii^	47.20 (7)
O3^ii^—K1—O1^vi^	109.72 (8)	N1—K3—O3^xii^	113.91 (5)
O3^iii^—K1—O1^vi^	111.53 (9)	O3^x^—K3—O3^xii^	104.69 (6)
O1^iv^—K1—O1^vi^	57.44 (9)	O3^xi^—K3—O3^xii^	104.69 (6)
O1^v^—K1—O1^vi^	57.44 (9)	O3^viii^—K3—Al1	129.57 (6)
O3^i^—K1—O2^iv^	63.69 (8)	O3^ix^—K3—Al1	129.57 (6)
O3^ii^—K1—O2^iv^	143.60 (8)	O3—K3—Al1	129.57 (6)
O3^iii^—K1—O2^iv^	94.55 (8)	O2^x^—K3—Al1	34.15 (6)
O1^iv^—K1—O2^iv^	48.34 (7)	O2^xi^—K3—Al1	34.15 (6)
O1^v^—K1—O2^iv^	81.13 (8)	O2^xii^—K3—Al1	34.15 (6)
O1^vi^—K1—O2^iv^	105.77 (8)	N1—K3—Al1	180.00 (9)
O3^i^—K1—O2^vi^	143.60 (8)	O3^x^—K3—Al1	66.09 (5)
O3^ii^—K1—O2^vi^	94.55 (8)	O3^xi^—K3—Al1	66.09 (5)
O3^iii^—K1—O2^vi^	63.69 (8)	O3^xii^—K3—Al1	66.09 (5)
O1^iv^—K1—O2^vi^	81.13 (8)	O3^viii^—K3—P1^xii^	168.66 (7)
O1^v^—K1—O2^vi^	105.77 (8)	O3^ix^—K3—P1^xii^	86.65 (6)
O1^vi^—K1—O2^vi^	48.34 (7)	O3—K3—P1^xii^	101.22 (6)
O2^iv^—K1—O2^vi^	115.34 (4)	O2^x^—K3—P1^xii^	82.96 (7)
O3^i^—K1—O2^v^	94.55 (8)	O2^xi^—K3—P1^xii^	64.96 (6)
O3^ii^—K1—O2^v^	63.69 (8)	O2^xii^—K3—P1^xii^	25.22 (6)
O3^iii^—K1—O2^v^	143.60 (8)	N1—K3—P1^xii^	125.75 (3)
O1^iv^—K1—O2^v^	105.77 (8)	O3^x^—K3—P1^xii^	79.71 (6)
O1^v^—K1—O2^v^	48.34 (7)	O3^xi^—K3—P1^xii^	112.01 (6)
O1^vi^—K1—O2^v^	81.13 (8)	O3^xii^—K3—P1^xii^	24.99 (5)
O2^iv^—K1—O2^v^	115.34 (4)	Al1—K3—P1^xii^	54.25 (3)
O2^vi^—K1—O2^v^	115.34 (4)	O1^xiii^—Al1—O1^xiv^	88.28 (14)
O3^i^—K1—Al1^vii^	132.11 (7)	O1^xiii^—Al1—O1^xv^	88.28 (14)
O3^ii^—K1—Al1^vii^	132.11 (7)	O1^xiv^—Al1—O1^xv^	88.28 (14)
O3^iii^—K1—Al1^vii^	132.11 (7)	O1^xiii^—Al1—O2^x^	92.92 (12)
O1^iv^—K1—Al1^vii^	33.70 (6)	O1^xiv^—Al1—O2^x^	175.81 (12)
O1^v^—K1—Al1^vii^	33.70 (6)	O1^xv^—Al1—O2^x^	87.74 (11)
O1^vi^—K1—Al1^vii^	33.70 (6)	O1^xiii^—Al1—O2^xi^	87.74 (11)
O2^iv^—K1—Al1^vii^	77.34 (5)	O1^xiv^—Al1—O2^xi^	92.92 (12)
O2^vi^—K1—Al1^vii^	77.34 (5)	O1^xv^—Al1—O2^xi^	175.81 (12)
O2^v^—K1—Al1^vii^	77.34 (5)	O2^x^—Al1—O2^xi^	91.14 (13)
O3^i^—K1—P1^iv^	88.98 (7)	O1^xiii^—Al1—O2^xii^	175.81 (12)
O3^ii^—K1—P1^iv^	168.77 (7)	O1^xiv^—Al1—O2^xii^	87.74 (11)
O3^iii^—K1—P1^iv^	100.08 (6)	O1^xv^—Al1—O2^xii^	92.92 (12)
O1^iv^—K1—P1^iv^	23.55 (6)	O2^x^—Al1—O2^xii^	91.14 (14)
O1^v^—K1—P1^iv^	70.37 (6)	O2^xi^—Al1—O2^xii^	91.14 (13)
O1^vi^—K1—P1^iv^	80.83 (7)	O1^xiii^—Al1—K3	126.47 (10)
O2^iv^—K1—P1^iv^	25.29 (5)	O1^xiv^—Al1—K3	126.47 (10)
O2^vi^—K1—P1^iv^	95.49 (6)	O1^xv^—Al1—K3	126.47 (10)
O2^v^—K1—P1^iv^	115.87 (6)	O2^x^—Al1—K3	55.54 (10)
Al1^vii^—K1—P1^iv^	55.54 (3)	O2^xi^—Al1—K3	55.54 (10)
O3^i^—K1—P1^vi^	168.77 (7)	O2^xii^—Al1—K3	55.54 (10)
O3^ii^—K1—P1^vi^	100.08 (6)	O1^xiii^—Al1—K1^xvi^	53.53 (10)
O3^iii^—K1—P1^vi^	88.98 (7)	O1^xiv^—Al1—K1^xvi^	53.53 (10)
O1^iv^—K1—P1^vi^	70.37 (6)	O1^xv^—Al1—K1^xvi^	53.53 (10)
O1^v^—K1—P1^vi^	80.83 (7)	O2^x^—Al1—K1^xvi^	124.46 (10)
O1^vi^—K1—P1^vi^	23.55 (6)	O2^xi^—Al1—K1^xvi^	124.46 (10)
O2^iv^—K1—P1^vi^	115.87 (6)	O2^xii^—Al1—K1^xvi^	124.46 (10)
O2^vi^—K1—P1^vi^	25.29 (5)	K3—Al1—K1^xvi^	180.00 (10)
O2^v^—K1—P1^vi^	95.49 (6)	O1^xiii^—Al1—K2^xv^	45.00 (8)
Al1^vii^—K1—P1^vi^	55.54 (3)	O1^xiv^—Al1—K2^xv^	125.19 (12)
P1^iv^—K1—P1^vi^	91.14 (4)	O1^xv^—Al1—K2^xv^	67.60 (8)
O3^iii^—K2—O3^i^	75.10 (9)	O2^x^—Al1—K2^xv^	54.13 (8)
O3^iii^—K2—O3^ii^	75.10 (9)	O2^xi^—Al1—K2^xv^	108.53 (8)
O3^i^—K2—O3^ii^	75.10 (9)	O2^xii^—Al1—K2^xv^	139.06 (9)
O3^iii^—K2—N1	135.27 (6)	K3—Al1—K2^xv^	106.68 (3)
O3^i^—K2—N1	135.27 (6)	K1^xvi^—Al1—K2^xv^	73.32 (3)
O3^ii^—K2—N1	135.27 (6)	O1^xiii^—Al1—K2^xvii^	67.60 (8)
O3^iii^—K2—O1^iii^	51.57 (8)	O1^xiv^—Al1—K2^xvii^	45.00 (8)
O3^i^—K2—O1^iii^	123.82 (8)	O1^xv^—Al1—K2^xvii^	125.19 (12)
O3^ii^—K2—O1^iii^	103.08 (8)	O2^x^—Al1—K2^xvii^	139.06 (9)
N1—K2—O1^iii^	85.66 (6)	O2^xi^—Al1—K2^xvii^	54.13 (8)
O3^iii^—K2—O1^i^	103.08 (8)	O2^xii^—Al1—K2^xvii^	108.53 (8)
O3^i^—K2—O1^i^	51.57 (8)	K3—Al1—K2^xvii^	106.68 (3)
O3^ii^—K2—O1^i^	123.82 (8)	K1^xvi^—Al1—K2^xvii^	73.32 (3)
N1—K2—O1^i^	85.66 (6)	K2^xv^—Al1—K2^xvii^	112.11 (3)
O1^iii^—K2—O1^i^	119.434 (16)	O1^xiii^—Al1—K2^xii^	125.19 (12)
O3^iii^—K2—O1^ii^	123.82 (8)	O1^xiv^—Al1—K2^xii^	67.60 (8)
O3^i^—K2—O1^ii^	103.08 (8)	O1^xv^—Al1—K2^xii^	45.00 (8)
O3^ii^—K2—O1^ii^	51.57 (8)	O2^x^—Al1—K2^xii^	108.53 (8)
N1—K2—O1^ii^	85.66 (6)	O2^xi^—Al1—K2^xii^	139.06 (9)
O1^iii^—K2—O1^ii^	119.434 (16)	O2^xii^—Al1—K2^xii^	54.13 (8)
O1^i^—K2—O1^ii^	119.434 (16)	K3—Al1—K2^xii^	106.68 (3)
O3^iii^—K2—O2	120.12 (8)	K1^xvi^—Al1—K2^xii^	73.32 (3)
O3^i^—K2—O2	155.11 (7)	K2^xv^—Al1—K2^xii^	112.11 (3)
O3^ii^—K2—O2	89.38 (7)	K2^xvii^—Al1—K2^xii^	112.11 (3)
N1—K2—O2	49.01 (5)	O3—P1—O1	111.58 (17)
O1^iii^—K2—O2	78.18 (7)	O3—P1—O2	113.45 (16)
O1^i^—K2—O2	131.74 (9)	O1—P1—O2	109.85 (16)
O1^ii^—K2—O2	52.43 (7)	O3—P1—N1	110.5 (2)
O3^iii^—K2—O2^viii^	89.38 (7)	O1—P1—N1	105.82 (13)
O3^i^—K2—O2^viii^	120.12 (8)	O2—P1—N1	105.20 (17)
O3^ii^—K2—O2^viii^	155.11 (7)	O3—P1—K2^xviii^	52.04 (12)
N1—K2—O2^viii^	49.01 (5)	O1—P1—K2^xviii^	60.00 (11)
O1^iii^—K2—O2^viii^	52.43 (7)	O2—P1—K2^xviii^	124.79 (11)
O1^i^—K2—O2^viii^	78.18 (7)	N1—P1—K2^xviii^	130.01 (13)
O1^ii^—K2—O2^viii^	131.74 (9)	O3—P1—K3^xix^	72.98 (11)
O2—K2—O2^viii^	81.64 (8)	O1—P1—K3^xix^	98.65 (11)
O3^iii^—K2—O2^ix^	155.11 (7)	O2—P1—K3^xix^	51.11 (11)
O3^i^—K2—O2^ix^	89.38 (7)	N1—P1—K3^xix^	151.24 (7)
O3^ii^—K2—O2^ix^	120.12 (8)	K2^xviii^—P1—K3^xix^	75.63 (3)
N1—K2—O2^ix^	49.01 (5)	O3—P1—K2	161.98 (12)
O1^iii^—K2—O2^ix^	131.74 (9)	O1—P1—K2	83.96 (11)
O1^i^—K2—O2^ix^	52.43 (7)	O2—P1—K2	66.87 (11)
O1^ii^—K2—O2^ix^	78.18 (7)	N1—P1—K2	54.41 (18)
O2—K2—O2^ix^	81.64 (8)	K2^xviii^—P1—K2	143.90 (4)
O2^viii^—K2—O2^ix^	81.64 (8)	K3^xix^—P1—K2	114.96 (3)
O3^iii^—K2—P1^iii^	25.20 (6)	O3—P1—K3	43.33 (11)
O3^i^—K2—P1^iii^	99.59 (7)	O1—P1—K3	113.68 (11)
O3^ii^—K2—P1^iii^	86.94 (7)	O2—P1—K3	136.05 (12)
N1—K2—P1^iii^	111.81 (3)	N1—P1—K3	68.57 (19)
O1^iii^—K2—P1^iii^	26.52 (6)	K2^xviii^—P1—K3	74.83 (3)
O1^i^—K2—P1^iii^	115.21 (6)	K3^xix^—P1—K3	114.91 (3)
O1^ii^—K2—P1^iii^	123.59 (6)	K2—P1—K3	122.98 (3)
O2—K2—P1^iii^	98.86 (5)	O3—P1—K1^xx^	119.94 (11)
O2^viii^—K2—P1^iii^	71.74 (5)	O1—P1—K1^xx^	46.13 (11)
O2^ix^—K2—P1^iii^	152.93 (6)	O2—P1—K1^xx^	65.25 (11)
O3^iii^—K2—P1^ii^	99.59 (7)	N1—P1—K1^xx^	128.30 (17)
O3^i^—K2—P1^ii^	86.94 (7)	K2^xviii^—P1—K1^xx^	78.82 (3)
O3^ii^—K2—P1^ii^	25.20 (6)	K3^xix^—P1—K1^xx^	61.97 (3)
N1—K2—P1^ii^	111.81 (3)	K2—P1—K1^xx^	77.22 (3)
O1^iii^—K2—P1^ii^	115.21 (6)	K3—P1—K1^xx^	153.19 (4)
O1^i^—K2—P1^ii^	123.59 (6)	P1^viii^—N1—P1^ix^	118.53 (8)
O1^ii^—K2—P1^ii^	26.52 (6)	P1^viii^—N1—P1	118.53 (8)
O2—K2—P1^ii^	71.74 (5)	P1^ix^—N1—P1	118.54 (8)
O2^viii^—K2—P1^ii^	152.93 (6)	P1^viii^—N1—K2	97.01 (18)
O2^ix^—K2—P1^ii^	98.86 (5)	P1^ix^—N1—K2	97.01 (18)
P1^iii^—K2—P1^ii^	107.04 (3)	P1—N1—K2	97.01 (18)
O3^viii^—K3—O3^ix^	83.76 (9)	P1^viii^—N1—K3	82.99 (18)
O3^viii^—K3—O3	83.76 (9)	P1^ix^—N1—K3	82.99 (18)
O3^ix^—K3—O3	83.76 (9)	P1—N1—K3	82.99 (18)
O3^viii^—K3—O2^x^	104.80 (8)	K2—N1—K3	180.0 (3)
O3^ix^—K3—O2^x^	162.69 (9)	P1—O1—Al1^xxi^	144.58 (18)
O3—K3—O2^x^	111.80 (8)	P1—O1—K1^xx^	110.32 (14)
O3^viii^—K3—O2^xi^	111.80 (8)	Al1^xxi^—O1—K1^xx^	92.77 (12)
O3^ix^—K3—O2^xi^	104.80 (8)	P1—O1—K2^xviii^	93.47 (13)
O3—K3—O2^xi^	162.69 (9)	Al1^xxi^—O1—K2^xviii^	108.00 (11)
O2^x^—K3—O2^xi^	58.18 (9)	K1^xx^—O1—K2^xviii^	101.99 (8)
O3^viii^—K3—O2^xii^	162.69 (9)	P1—O2—Al1^xix^	131.82 (18)
O3^ix^—K3—O2^xii^	111.80 (8)	P1—O2—K3^xix^	103.68 (13)
O3—K3—O2^xii^	104.80 (8)	Al1^xix^—O2—K3^xix^	90.31 (11)
O2^x^—K3—O2^xii^	58.18 (9)	P1—O2—K1^xx^	89.45 (12)
O2^xi^—K3—O2^xii^	58.18 (9)	Al1^xix^—O2—K1^xx^	138.56 (12)
O3^viii^—K3—N1	50.43 (6)	K3^xix^—O2—K1^xx^	73.60 (7)
O3^ix^—K3—N1	50.43 (6)	P1—O2—K2	87.50 (12)
O3—K3—N1	50.43 (6)	Al1^xix^—O2—K2	97.57 (11)
O2^x^—K3—N1	145.85 (6)	K3^xix^—O2—K2	156.49 (11)
O2^xi^—K3—N1	145.85 (6)	K1^xx^—O2—K2	86.08 (7)
O2^xii^—K3—N1	145.85 (6)	P1—O3—K1^xviii^	153.19 (17)
O3^viii^—K3—O3^x^	111.63 (2)	P1—O3—K3	114.19 (14)
O3^ix^—K3—O3^x^	143.68 (7)	K1^xviii^—O3—K3	87.05 (9)
O3—K3—O3^x^	66.44 (10)	P1—O3—K2^xviii^	102.76 (15)
O2^x^—K3—O3^x^	47.20 (7)	K1^xviii^—O3—K2^xviii^	87.38 (8)
O2^xi^—K3—O3^x^	99.58 (8)	K3—O3—K2^xviii^	102.41 (10)
O2^xii^—K3—O3^x^	60.45 (8)	P1—O3—K3^xix^	82.03 (12)
N1—K3—O3^x^	113.91 (5)	K1^xviii^—O3—K3^xix^	73.74 (6)
O3^viii^—K3—O3^xi^	66.44 (10)	K3—O3—K3^xix^	158.41 (11)
O3^ix^—K3—O3^xi^	111.63 (2)	K2^xviii^—O3—K3^xix^	86.76 (7)
O3—K3—O3^xi^	143.68 (7)		

Symmetry codes: (i) −*y*+1, *z*−1/2, −*x*+1/2; (ii) −*x*+1/2, −*y*+1, *z*−1/2; (iii) *z*−1/2, −*x*+1/2, −*y*+1; (iv) −*z*+1/2, −*x*, *y*−1/2; (v) *y*−1/2, −*z*+1/2, −*x*; (vi) −*x*, *y*−1/2, −*z*+1/2; (vii) *x*−1, *y*−1, *z*−1; (viii) *z*, *x*, *y*; (ix) *y*, *z*, *x*; (x) −*z*+1, *x*+1/2, −*y*+3/2; (xi) −*y*+3/2, −*z*+1, *x*+1/2; (xii) *x*+1/2, −*y*+3/2, −*z*+1; (xiii) −*z*+3/2, −*x*+1, *y*+1/2; (xiv) *y*+1/2, −*z*+3/2, −*x*+1; (xv) −*x*+1, *y*+1/2, −*z*+3/2; (xvi) *x*+1, *y*+1, *z*+1; (xvii) −*x*+3/2, −*y*+1, *z*+1/2; (xviii) −*x*+1/2, −*y*+1, *z*+1/2; (xix) *x*−1/2, −*y*+3/2, −*z*+1; (xx) −*x*, *y*+1/2, −*z*+1/2; (xxi) −*x*+1, *y*−1/2, −*z*+3/2.

### Tripotassium gallium nitridotriphosphate (II) Crystal data

**Table:**

K_3_Ga(NP_3_O_9_)	*D*_x_ = 3.157 Mg m^−^^3^
*M**_r_* = 437.94	Mo *K*α radiation, λ = 0.71073 Å
Cubic, *P*2_1_3	Cell parameters from 4964 reflections
Hall symbol: P 2ac 2ab 3	θ = 3.0–28.0°
*a* = 9.7313 (9) Å	µ = 4.90 mm^−^^1^
*V* = 921.5 (3) Å^3^	*T* = 293 K
*Z* = 4	Irregular tetrahedron, colorless
*F*(000) = 848	0.12 × 0.07 × 0.04 mm

### Tripotassium gallium nitridotriphosphate (II) Data collection

**Table:**

Oxford Diffraction Xcalibur-3 diffractometer	517 reflections with *I* > 2σ(*I*)
Graphite monochromator	*R*_int_ = 0.155
φ and ω scans	θ_max_ = 28.0°, θ_min_ = 3.0°
Absorption correction: multi-scan (Blessing, 1995)	*h* = −12→9
*T*_min_ = 0.738, *T*_max_ = 0.804	*k* = −12→9
4964 measured reflections	*l* = −10→12
753 independent reflections	

### Tripotassium gallium nitridotriphosphate (II) Refinement

**Table:**

Refinement on *F*^2^	'*w* = 1/[σ^2^(*F*_o_^2^) + (0.0188*P*)^2^] where *P* = (*F*_o_^2^ + 2*F*_c_^2^)/3'
Least-squares matrix: full	(Δ/σ)_max_ < 0.001
*R*[*F*^2^ > 2σ(*F*^2^)] = 0.059	Δρ_max_ = 0.67 e Å^−^^3^
*wR*(*F*^2^) = 0.102	Δρ_min_ = −0.60 e Å^−^^3^
*S* = 1	Extinction correction: SHELXL2018/3 (Sheldrick 2015)
753 reflections	Extinction coefficient: 0.0038 (15)
53 parameters	Absolute structure: Flack *x* determined using 153 quotients [(*I*^+^)-(*I*^-^)]/[(*I*^+^)+(*I*^-^)] (Parsons et al., 2013)
0 restraints	Absolute structure parameter: 0.03 (5)

### Tripotassium gallium nitridotriphosphate (II) Special details

**Table:**

Geometry. All esds (except the esd in the dihedral angle between two l.s. planes) are estimated using the full covariance matrix. The cell esds are taken into account individually in the estimation of esds in distances, angles and torsion angles; correlations between esds in cell parameters are only used when they are defined by crystal symmetry. An approximate (isotropic) treatment of cell esds is used for estimating esds involving l.s. planes.

### Tripotassium gallium nitridotriphosphate (II) Fractional atomic coordinates and isotropic or equivalent isotropic displacement parameters (Å^2^)

**Table:**

	*x*	*y*	*z*	*U*_iso_*/*U*_eq_	
K1	0.0456 (3)	0.0456 (3)	0.0456 (3)	0.0326 (13)	
K2	0.2679 (3)	0.2679 (3)	0.2679 (3)	0.0329 (13)	
K3	0.6410 (3)	0.6410 (3)	0.6410 (3)	0.0358 (14)	
Ga1	0.83974 (15)	0.83974 (15)	0.83974 (15)	0.0235 (7)	
P1	0.3255 (4)	0.5696 (3)	0.4719 (3)	0.0221 (8)	
N1	0.4446 (10)	0.4446 (10)	0.4446 (10)	0.019 (4)	
O1	0.2172 (8)	0.5088 (9)	0.5672 (8)	0.024 (2)	
O2	0.2578 (8)	0.5989 (8)	0.3347 (9)	0.026 (2)	
O3	0.3941 (8)	0.6899 (8)	0.5330 (8)	0.028 (2)	

### Tripotassium gallium nitridotriphosphate (II) Atomic displacement parameters (Å^2^)

**Table:**

	*U*^11^	*U*^22^	*U*^33^	*U*^12^	*U*^13^	*U*^23^
K1	0.0326 (13)	0.0326 (13)	0.0326 (13)	−0.0026 (14)	−0.0026 (14)	−0.0026 (14)
K2	0.0329 (13)	0.0329 (13)	0.0329 (13)	−0.0034 (14)	−0.0034 (14)	−0.0034 (14)
K3	0.0358 (14)	0.0358 (14)	0.0358 (14)	−0.0040 (16)	−0.0040 (16)	−0.0040 (16)
Ga1	0.0235 (7)	0.0235 (7)	0.0235 (7)	0.0007 (7)	0.0007 (7)	0.0007 (7)
P1	0.0243 (18)	0.0217 (18)	0.0204 (17)	−0.0025 (15)	−0.0006 (15)	−0.0001 (14)
N1	0.019 (4)	0.019 (4)	0.019 (4)	0.003 (5)	0.003 (5)	0.003 (5)
O1	0.022 (5)	0.029 (5)	0.021 (5)	−0.002 (4)	0.007 (4)	0.010 (4)
O2	0.022 (4)	0.031 (5)	0.026 (5)	0.005 (4)	−0.001 (4)	−0.003 (4)
O3	0.039 (6)	0.023 (5)	0.023 (5)	−0.005 (4)	−0.002 (4)	−0.006 (4)

### Tripotassium gallium nitridotriphosphate (II) Geometric parameters (Å, º)

**Table:**

K1—O3^i^	2.642 (8)	K2—P1^i^	3.409 (4)
K1—O3^ii^	2.642 (8)	K3—O3	2.665 (9)
K1—O3^iii^	2.642 (8)	K3—O3^viii^	2.665 (9)
K1—O1^iv^	2.807 (9)	K3—O3^ix^	2.665 (9)
K1—O1^v^	2.807 (9)	K3—O2^x^	2.785 (8)
K1—O1^vi^	2.807 (9)	K3—O2^xi^	2.785 (8)
K1—O2^v^	3.216 (8)	K3—O2^xii^	2.785 (8)
K1—O2^vi^	3.216 (8)	K3—N1	3.310 (18)
K1—O2^iv^	3.216 (8)	K3—Ga1	3.350 (5)
K1—Ga1^vii^	3.470 (6)	K3—O3^x^	3.412 (8)
K1—P1^iv^	3.624 (5)	K3—O3^xi^	3.412 (8)
K1—P1^vi^	3.624 (5)	K3—O3^xii^	3.412 (8)
K2—O3^ii^	2.808 (9)	K3—P1^xii^	3.516 (4)
K2—O3^i^	2.808 (9)	Ga1—O1^xiii^	1.958 (8)
K2—O3^iii^	2.808 (9)	Ga1—O1^xiv^	1.958 (8)
K2—O1^i^	2.925 (9)	Ga1—O1^xv^	1.958 (8)
K2—O1^ii^	2.925 (9)	Ga1—O2^xi^	1.968 (9)
K2—O1^iii^	2.925 (9)	Ga1—O2^x^	1.968 (9)
K2—N1	2.977 (18)	Ga1—O2^xii^	1.968 (9)
K2—O2^viii^	3.287 (8)	P1—O3	1.473 (9)
K2—O2^ix^	3.287 (8)	P1—O2	1.516 (9)
K2—O2	3.287 (8)	P1—O1	1.524 (9)
K2—P1^ii^	3.409 (4)	P1—N1	1.701 (4)
			
O3^i^—K1—O3^ii^	80.8 (3)	O2^x^—K3—O3^x^	46.3 (2)
O3^i^—K1—O3^iii^	80.8 (3)	O2^xi^—K3—O3^x^	101.1 (2)
O3^ii^—K1—O3^iii^	80.8 (3)	O2^xii^—K3—O3^x^	60.3 (2)
O3^i^—K1—O1^iv^	111.5 (2)	N1—K3—O3^x^	114.13 (16)
O3^ii^—K1—O1^iv^	165.6 (3)	Ga1—K3—O3^x^	65.87 (16)
O3^iii^—K1—O1^iv^	108.0 (3)	O3—K3—O3^xi^	143.91 (19)
O3^i^—K1—O1^v^	108.0 (3)	O3^viii^—K3—O3^xi^	111.65 (7)
O3^ii^—K1—O1^v^	111.5 (2)	O3^ix^—K3—O3^xi^	67.3 (3)
O3^iii^—K1—O1^v^	165.6 (3)	O2^x^—K3—O3^xi^	60.3 (2)
O1^iv^—K1—O1^v^	58.5 (3)	O2^xi^—K3—O3^xi^	46.3 (2)
O3^i^—K1—O1^vi^	165.6 (3)	O2^xii^—K3—O3^xi^	101.1 (2)
O3^ii^—K1—O1^vi^	108.0 (3)	N1—K3—O3^xi^	114.13 (16)
O3^iii^—K1—O1^vi^	111.5 (2)	Ga1—K3—O3^xi^	65.87 (16)
O1^iv^—K1—O1^vi^	58.5 (3)	O3^x^—K3—O3^xi^	104.44 (18)
O1^v^—K1—O1^vi^	58.5 (3)	O3—K3—O3^xii^	111.65 (7)
O3^i^—K1—O2^v^	93.7 (2)	O3^viii^—K3—O3^xii^	67.3 (3)
O3^ii^—K1—O2^v^	64.5 (2)	O3^ix^—K3—O3^xii^	143.91 (19)
O3^iii^—K1—O2^v^	145.2 (3)	O2^x^—K3—O3^xii^	101.1 (2)
O1^iv^—K1—O2^v^	106.0 (3)	O2^xi^—K3—O3^xii^	60.3 (2)
O1^v^—K1—O2^v^	47.6 (2)	O2^xii^—K3—O3^xii^	46.3 (2)
O1^vi^—K1—O2^v^	80.4 (2)	N1—K3—O3^xii^	114.13 (16)
O3^i^—K1—O2^vi^	145.2 (3)	Ga1—K3—O3^xii^	65.87 (16)
O3^ii^—K1—O2^vi^	93.7 (2)	O3^x^—K3—O3^xii^	104.44 (18)
O3^iii^—K1—O2^vi^	64.5 (2)	O3^xi^—K3—O3^xii^	104.44 (18)
O1^iv^—K1—O2^vi^	80.4 (2)	O3—K3—P1^xii^	101.2 (2)
O1^v^—K1—O2^vi^	106.0 (3)	O3^viii^—K3—P1^xii^	86.72 (18)
O1^vi^—K1—O2^vi^	47.6 (2)	O3^ix^—K3—P1^xii^	168.3 (2)
O2^v^—K1—O2^vi^	114.96 (13)	O2^x^—K3—P1^xii^	85.1 (2)
O3^i^—K1—O2^iv^	64.5 (2)	O2^xi^—K3—P1^xii^	66.1 (2)
O3^ii^—K1—O2^iv^	145.2 (3)	O2^xii^—K3—P1^xii^	24.51 (18)
O3^iii^—K1—O2^iv^	93.7 (2)	N1—K3—P1^xii^	125.23 (9)
O1^iv^—K1—O2^iv^	47.6 (2)	Ga1—K3—P1^xii^	54.77 (9)
O1^v^—K1—O2^iv^	80.4 (2)	O3^x^—K3—P1^xii^	79.98 (17)
O1^vi^—K1—O2^iv^	106.0 (3)	O3^xi^—K3—P1^xii^	112.13 (19)
O2^v^—K1—O2^iv^	114.96 (13)	O3^xii^—K3—P1^xii^	24.49 (15)
O2^vi^—K1—O2^iv^	114.96 (13)	O1^xiii^—Ga1—O1^xiv^	88.9 (4)
O3^i^—K1—Ga1^vii^	131.6 (2)	O1^xiii^—Ga1—O1^xv^	88.9 (4)
O3^ii^—K1—Ga1^vii^	131.6 (2)	O1^xiv^—Ga1—O1^xv^	88.9 (4)
O3^iii^—K1—Ga1^vii^	131.6 (2)	O1^xiii^—Ga1—O2^xi^	91.7 (3)
O1^iv^—K1—Ga1^vii^	34.33 (17)	O1^xiv^—Ga1—O2^xi^	176.3 (3)
O1^v^—K1—Ga1^vii^	34.33 (17)	O1^xv^—Ga1—O2^xi^	87.5 (3)
O1^vi^—K1—Ga1^vii^	34.33 (17)	O1^xiii^—Ga1—O2^x^	176.3 (3)
O2^v^—K1—Ga1^vii^	76.82 (17)	O1^xiv^—Ga1—O2^x^	87.5 (3)
O2^vi^—K1—Ga1^vii^	76.82 (17)	O1^xv^—Ga1—O2^x^	91.7 (4)
O2^iv^—K1—Ga1^vii^	76.82 (17)	O2^xi^—Ga1—O2^x^	92.0 (3)
O3^i^—K1—P1^iv^	89.16 (19)	O1^xiii^—Ga1—O2^xii^	87.5 (3)
O3^ii^—K1—P1^iv^	169.8 (2)	O1^xiv^—Ga1—O2^xii^	91.7 (3)
O3^iii^—K1—P1^iv^	98.99 (18)	O1^xv^—Ga1—O2^xii^	176.3 (3)
O1^iv^—K1—P1^iv^	23.26 (16)	O2^xi^—Ga1—O2^xii^	92.0 (3)
O1^v^—K1—P1^iv^	70.29 (19)	O2^x^—Ga1—O2^xii^	92.0 (3)
O1^vi^—K1—P1^iv^	81.7 (2)	O1^xiii^—Ga1—K3	126.1 (2)
O2^v^—K1—P1^iv^	115.3 (2)	O1^xiv^—Ga1—K3	126.1 (2)
O2^vi^—K1—P1^iv^	95.39 (17)	O1^xv^—Ga1—K3	126.1 (2)
O2^iv^—K1—P1^iv^	24.71 (16)	O2^xi^—Ga1—K3	56.2 (2)
Ga1^vii^—K1—P1^iv^	55.56 (9)	O2^x^—Ga1—K3	56.2 (2)
O3^i^—K1—P1^vi^	169.8 (2)	O2^xii^—Ga1—K3	56.2 (2)
O3^ii^—K1—P1^vi^	98.99 (18)	O1^xiii^—Ga1—K1^xvi^	53.9 (2)
O3^iii^—K1—P1^vi^	89.16 (19)	O1^xiv^—Ga1—K1^xvi^	53.9 (2)
O1^iv^—K1—P1^vi^	70.29 (19)	O1^xv^—Ga1—K1^xvi^	53.9 (2)
O1^v^—K1—P1^vi^	81.7 (2)	O2^xi^—Ga1—K1^xvi^	123.8 (2)
O1^vi^—K1—P1^vi^	23.26 (17)	O2^x^—Ga1—K1^xvi^	123.8 (2)
O2^v^—K1—P1^vi^	95.39 (17)	O2^xii^—Ga1—K1^xvi^	123.8 (2)
O2^vi^—K1—P1^vi^	24.71 (16)	K3—Ga1—K1^xvi^	180.00 (7)
O2^iv^—K1—P1^vi^	115.3 (2)	O1^xiii^—Ga1—K2^xiv^	124.6 (3)
Ga1^vii^—K1—P1^vi^	55.56 (9)	O1^xiv^—Ga1—K2^xiv^	68.5 (3)
P1^iv^—K1—P1^vi^	91.16 (13)	O1^xv^—Ga1—K2^xiv^	43.4 (3)
O3^ii^—K2—O3^i^	75.1 (3)	O2^xi^—Ga1—K2^xiv^	108.3 (2)
O3^ii^—K2—O3^iii^	75.1 (3)	O2^x^—Ga1—K2^xiv^	54.3 (2)
O3^i^—K2—O3^iii^	75.1 (3)	O2^xii^—Ga1—K2^xiv^	140.0 (2)
O3^ii^—K2—O1^i^	123.7 (3)	K3—Ga1—K2^xiv^	107.30 (4)
O3^i^—K2—O1^i^	51.4 (2)	K1^xvi^—Ga1—K2^xiv^	72.70 (4)
O3^iii^—K2—O1^i^	102.9 (2)	O1^xiii^—Ga1—K2^xvii^	43.4 (3)
O3^ii^—K2—O1^ii^	51.4 (2)	O1^xiv^—Ga1—K2^xvii^	124.6 (3)
O3^i^—K2—O1^ii^	102.9 (2)	O1^xv^—Ga1—K2^xvii^	68.5 (3)
O3^iii^—K2—O1^ii^	123.7 (3)	O2^xi^—Ga1—K2^xvii^	54.3 (2)
O1^i^—K2—O1^ii^	119.49 (5)	O2^x^—Ga1—K2^xvii^	140.0 (2)
O3^ii^—K2—O1^iii^	102.9 (2)	O2^xii^—Ga1—K2^xvii^	108.3 (2)
O3^i^—K2—O1^iii^	123.7 (3)	K3—Ga1—K2^xvii^	107.30 (4)
O3^iii^—K2—O1^iii^	51.4 (2)	K1^xvi^—Ga1—K2^xvii^	72.70 (4)
O1^i^—K2—O1^iii^	119.49 (5)	K2^xiv^—Ga1—K2^xvii^	111.55 (3)
O1^ii^—K2—O1^iii^	119.49 (5)	O1^xiii^—Ga1—K2^xii^	68.5 (3)
O3^ii^—K2—N1	135.25 (18)	O1^xiv^—Ga1—K2^xii^	43.4 (3)
O3^i^—K2—N1	135.25 (18)	O1^xv^—Ga1—K2^xii^	124.6 (3)
O3^iii^—K2—N1	135.25 (18)	O2^xi^—Ga1—K2^xii^	140.0 (2)
O1^i^—K2—N1	85.89 (18)	O2^x^—Ga1—K2^xii^	108.3 (2)
O1^ii^—K2—N1	85.89 (18)	O2^xii^—Ga1—K2^xii^	54.3 (2)
O1^iii^—K2—N1	85.89 (18)	K3—Ga1—K2^xii^	107.30 (4)
O3^ii^—K2—O2^viii^	90.1 (2)	K1^xvi^—Ga1—K2^xii^	72.70 (4)
O3^i^—K2—O2^viii^	156.0 (2)	K2^xiv^—Ga1—K2^xii^	111.55 (3)
O3^iii^—K2—O2^viii^	119.8 (2)	K2^xvii^—Ga1—K2^xii^	111.55 (3)
O1^i^—K2—O2^viii^	131.8 (3)	O3—P1—O2	113.8 (5)
O1^ii^—K2—O2^viii^	53.5 (2)	O3—P1—O1	112.1 (5)
O1^iii^—K2—O2^viii^	77.6 (2)	O2—P1—O1	107.9 (5)
N1—K2—O2^viii^	48.52 (15)	O3—P1—N1	108.9 (7)
O3^ii^—K2—O2^ix^	119.8 (2)	O2—P1—N1	107.0 (5)
O3^i^—K2—O2^ix^	90.1 (2)	O1—P1—N1	106.8 (4)
O3^iii^—K2—O2^ix^	156.0 (2)	O3—P1—K2^xviii^	53.9 (4)
O1^i^—K2—O2^ix^	53.5 (2)	O2—P1—K2^xviii^	122.8 (3)
O1^ii^—K2—O2^ix^	77.6 (2)	O1—P1—K2^xviii^	58.8 (4)
O1^iii^—K2—O2^ix^	131.8 (3)	N1—P1—K2^xviii^	130.2 (4)
N1—K2—O2^ix^	48.52 (15)	O3—P1—K3^xix^	73.8 (3)
O2^viii^—K2—O2^ix^	80.9 (2)	O2—P1—K3^xix^	49.6 (3)
O3^ii^—K2—O2	156.0 (2)	O1—P1—K3^xix^	98.5 (3)
O3^i^—K2—O2	119.8 (2)	N1—P1—K3^xix^	150.7 (2)
O3^iii^—K2—O2	90.1 (2)	K2^xviii^—P1—K3^xix^	75.90 (10)
O1^i^—K2—O2	77.6 (2)	O3—P1—K3	42.8 (3)
O1^ii^—K2—O2	131.8 (3)	O2—P1—K3	138.4 (3)
O1^iii^—K2—O2	53.5 (2)	O1—P1—K3	113.1 (3)
N1—K2—O2	48.52 (15)	N1—P1—K3	67.9 (6)
O2^viii^—K2—O2	80.9 (2)	K2^xviii^—P1—K3	75.44 (8)
O2^ix^—K2—O2	80.9 (2)	K3^xix^—P1—K3	115.45 (9)
O3^ii^—K2—P1^ii^	25.10 (18)	O3—P1—K2	160.8 (4)
O3^i^—K2—P1^ii^	86.7 (2)	O2—P1—K2	66.3 (3)
O3^iii^—K2—P1^ii^	99.6 (2)	O1—P1—K2	84.9 (4)
O1^i^—K2—P1^ii^	123.33 (17)	N1—P1—K2	55.6 (6)
O1^ii^—K2—P1^ii^	26.44 (17)	K2^xviii^—P1—K2	143.62 (12)
O1^iii^—K2—P1^ii^	115.25 (16)	K3^xix^—P1—K2	113.74 (10)
N1—K2—P1^ii^	111.98 (9)	K3—P1—K2	123.46 (11)
O2^viii^—K2—P1^ii^	72.82 (16)	O3—P1—K1^xx^	121.5 (3)
O2^ix^—K2—P1^ii^	98.30 (15)	O2—P1—K1^xx^	62.4 (3)
O2—K2—P1^ii^	153.45 (17)	O1—P1—K1^xx^	46.7 (3)
O3^ii^—K2—P1^i^	99.6 (2)	N1—P1—K1^xx^	128.7 (6)
O3^i^—K2—P1^i^	25.10 (18)	K2^xviii^—P1—K1^xx^	78.68 (8)
O3^iii^—K2—P1^i^	86.7 (2)	K3^xix^—P1—K1^xx^	61.82 (9)
O1^i^—K2—P1^i^	26.44 (17)	K3—P1—K1^xx^	153.65 (11)
O1^ii^—K2—P1^i^	115.25 (16)	K2—P1—K1^xx^	76.43 (8)
O1^iii^—K2—P1^i^	123.33 (17)	P1—N1—P1^viii^	118.8 (2)
N1—K2—P1^i^	111.98 (9)	P1—N1—P1^ix^	118.8 (2)
O2^viii^—K2—P1^i^	153.45 (17)	P1^viii^—N1—P1^ix^	118.8 (2)
O2^ix^—K2—P1^i^	72.82 (16)	P1—N1—K2	96.3 (6)
O2—K2—P1^i^	98.30 (15)	P1^viii^—N1—K2	96.3 (6)
P1^ii^—K2—P1^i^	106.85 (10)	P1^ix^—N1—K2	96.3 (6)
O3—K3—O3^viii^	82.9 (3)	P1—N1—K3	83.7 (6)
O3—K3—O3^ix^	82.9 (3)	P1^viii^—N1—K3	83.7 (6)
O3^viii^—K3—O3^ix^	82.9 (3)	P1^ix^—N1—K3	83.7 (6)
O3—K3—O2^x^	111.2 (2)	K2—N1—K3	180.0 (9)
O3^viii^—K3—O2^x^	164.8 (3)	P1—O1—Ga1^xxi^	143.5 (6)
O3^ix^—K3—O2^x^	103.8 (2)	P1—O1—K1^xx^	110.0 (4)
O3—K3—O2^xi^	164.8 (3)	Ga1^xxi^—O1—K1^xx^	91.7 (3)
O3^viii^—K3—O2^xi^	103.8 (2)	P1—O1—K2^xviii^	94.8 (4)
O3^ix^—K3—O2^xi^	111.2 (2)	Ga1^xxi^—O1—K2^xviii^	109.2 (3)
O2^x^—K3—O2^xi^	61.1 (3)	K1^xx^—O1—K2^xviii^	102.2 (3)
O3—K3—O2^xii^	103.8 (2)	P1—O2—Ga1^xix^	129.8 (5)
O3^viii^—K3—O2^xii^	111.2 (2)	P1—O2—K3^xix^	105.8 (4)
O3^ix^—K3—O2^xii^	164.8 (3)	Ga1^xix^—O2—K3^xix^	87.9 (3)
O2^x^—K3—O2^xii^	61.1 (3)	P1—O2—K1^xx^	92.9 (4)
O2^xi^—K3—O2^xii^	61.1 (3)	Ga1^xix^—O2—K1^xx^	137.2 (4)
O3—K3—N1	49.85 (19)	K3^xix^—O2—K1^xx^	74.99 (18)
O3^viii^—K3—N1	49.85 (19)	P1—O2—K2	88.7 (3)
O3^ix^—K3—N1	49.85 (19)	Ga1^xix^—O2—K2	96.6 (3)
O2^x^—K3—N1	144.05 (18)	K3^xix^—O2—K2	156.9 (3)
O2^xi^—K3—N1	144.05 (18)	K1^xx^—O2—K2	86.6 (2)
O2^xii^—K3—N1	144.05 (18)	P1—O3—K1^xviii^	153.3 (5)
O3—K3—Ga1	130.15 (19)	P1—O3—K3	115.2 (4)
O3^viii^—K3—Ga1	130.15 (19)	K1^xviii^—O3—K3	87.5 (3)
O3^ix^—K3—Ga1	130.15 (19)	P1—O3—K2^xviii^	101.0 (4)
O2^x^—K3—Ga1	35.95 (18)	K1^xviii^—O3—K2^xviii^	86.8 (3)
O2^xi^—K3—Ga1	35.95 (18)	K3—O3—K2^xviii^	102.2 (3)
O2^xii^—K3—Ga1	35.95 (18)	P1—O3—K3^xix^	81.7 (3)
N1—K3—Ga1	180.0 (3)	K1^xviii^—O3—K3^xix^	73.36 (19)
O3—K3—O3^x^	67.3 (3)	K3—O3—K3^xix^	158.9 (3)
O3^viii^—K3—O3^x^	143.91 (19)	K2^xviii^—O3—K3^xix^	85.9 (2)
O3^ix^—K3—O3^x^	111.65 (7)		

Symmetry codes: (i) *z*−1/2, −*x*+1/2, −*y*+1; (ii) −*y*+1, *z*−1/2, −*x*+1/2; (iii) −*x*+1/2, −*y*+1, *z*−1/2; (iv) −*x*, *y*−1/2, −*z*+1/2; (v) −*z*+1/2, −*x*, *y*−1/2; (vi) *y*−1/2, −*z*+1/2, −*x*; (vii) *x*−1, *y*−1, *z*−1; (viii) *y*, *z*, *x*; (ix) *z*, *x*, *y*; (x) −*z*+1, *x*+1/2, −*y*+3/2; (xi) −*y*+3/2, −*z*+1, *x*+1/2; (xii) *x*+1/2, −*y*+3/2, −*z*+1; (xiii) *y*+1/2, −*z*+3/2, −*x*+1; (xiv) −*x*+1, *y*+1/2, −*z*+3/2; (xv) −*z*+3/2, −*x*+1, *y*+1/2; (xvi) *x*+1, *y*+1, *z*+1; (xvii) −*x*+3/2, −*y*+1, *z*+1/2; (xviii) −*x*+1/2, −*y*+1, *z*+1/2; (xix) *x*−1/2, −*y*+3/2, −*z*+1; (xx) −*x*, *y*+1/2, −*z*+1/2; (xxi) −*x*+1, *y*−1/2, −*z*+3/2.
